# Laugh-induced seizure: a case report

**DOI:** 10.1186/1752-1947-7-123

**Published:** 2013-05-13

**Authors:** Naba Raj Mainali, Leena Jalota, Madan Raj Aryal, Torrey R Schmidt, Madan Badal, Richard Alweis

**Affiliations:** 1 Department of Medicine, Reading Health System, Sixth Avenue and Spruce Street, West Reading, PA, 19611, USA; 2 Department of Medicine, Robert Wood Johnson University Hospital, 10 Plum Street, New Brunswick, NJ, 08901, USA; 3 Jefferson Medical College, 1025 Walnut Street, Philadelphia, PA, 19107, USA

**Keywords:** Laugh-induced seizure, Gelastic seizure, EEG, Cingulate gyrus, Topiramate, Carbamazepine

## Abstract

**Introduction:**

A laugh-induced seizure is an unrecognized condition and to the best of our knowledge no case has been reported in the medical literature until now*.* We present an interesting and extremely rare case in which laughing generated the seizure activity that was recorded and confirmed by video electroencephalography.

**Case presentation:**

A 43-year-old obese Caucasian man with history of bipolar disorder and chronic headache presented with multiple episodes of seizures, all induced by laughter while watching comedy shows. Each episode lasted approximately five seconds. In each instance, he started laughing, then his arms started shaking and he felt like ‘his consciousness was being vacuumed away’. A physical examination revealed normal findings. He had been maintained on valproic acid for bipolar disorder and topiramate for his chronic headache, but this did not control his symptoms. His sleep-deprived electroencephalography and brain magnetic resonance imaging were normal except for an arachnoid cyst measuring 4.2 × 2.1cm in the anterior right middle cranial fossa. His video electroencephalography demonstrated laugh-induced seizure activities. He was then placed on carbamazepine. Following treatment, he had two episodes of mild staring but no frank seizures, and his seizures have remained well controlled on this regimen for more than a year.

**Conclusions:**

Laugh-induced seizure is a most unusual clinical entity without any previous case report. Confirmatory diagnosis can be made by video electroencephalography recording of seizure activities provoked by laughing. As in gelastic seizure without hypothalamic hamartoma, our case responded well to polytherapy with topiramate and carbamazepine on top of laugh-provocation avoidance. Further study is required to establish the standard treatment of this condition.

## Introduction

Laughing is an entirely human quality and a part of everyday life. Laughter has a positive effect on measures of generalized well-being [1]. Laugh-induced seizure is an extremely rare and probably unrecognized condition and thus can easily be misdiagnosed. However, it is very important to recognize this condition as early diagnosis and treatment may control the seizure activities and improve quality of life. To the best of our knowledge, no case of laugh-induced seizure has been reported in the medical literature. Based upon our case report, we discuss the fundamentals of this condition including clinical presentation, diagnostic modalities and possible treatment options, and differentiating features with the more common condition, the gelastic seizure. Seizure activities are induced by laughter in laugh-induced seizure but laughter is the manifestation of gelastic seizure.

## Case presentation

A 43-year-old obese, Caucasian man with a past history of insomnia, bipolar disorder and chronic headache presented with multiple episodes of seizures; all were induced by laughter. He had had several mild seizures in the month prior to admission, induced by laughter when he was watching comedy shows on the television. Each episode lasted approximately five to 10 seconds. In each instance, he started laughing, then his arms started shaking and he felt like ‘his consciousness was being vacuumed away’. He denied any history of tongue bite, bowel or bladder incontinence. He had had a variable number of seizure episodes; five times a day on average, based upon the length and intensity of the comedy shows. There was no family history of seizure disorder in his first-degree relatives. A physical examination revealed an alert, awake obese man with stable vital signs including blood pressure of 123/67mm Hg and a regular heart rate of 75 per minute. His respiratory and cardiovascular examinations were within normal limits. A neurological examination revealed intact cranial nerves, normal motor, sensory and cerebellar functions with no focal neurological deficits.

All of his electrolytes including sodium, magnesium, and calcium were within normal range. Glucose was also within normal range. His sleep-deprived electroencephalography (EEG) was normal and a brain magnetic resonance imaging (MRI) scan revealed an arachnoid cyst measuring 4.2 × 2.1cm in the anterior right middle cranial fossa. He was then admitted for a two-day video EEG monitoring, which revealed several bursts of generalized high-amplitude spike and wave activity with frontotemporal predominance, associated with staring episodes, rolling up of the eyes, unresponsiveness to questions and tonic-clonic activities for five to 10 seconds, provoked by ‘deep belly’ laughing. He reported complete lack of awareness during those episodes.

All those manifestations were initially considered to be due to bipolar disorder and he had been maintained on valproic acid and topiramate for his chronic headaches, but this did not seem to be controlling his symptoms. Other differentials included drug-induced seizures, mainly secondary to valproic acid, which was unlikely as the drug levels were normal. In addition, the patient continued to have seizure episodes even during the period of time he was off valproic acid. Conversion disorder was also considered but a two-day video EEG did reveal high-amplitude spikes. Another diagnosis considered was increased intracranial pressure secondary to the arachnoid cyst, leading to compression of the right temporal lobe, triggering temporal lobe epilepsy. However, the video EEG did not display any discrete temporal lobe activities and his ventricles were normal sized on both computed tomography and MRI imaging of his brain. He never had seizure activities without the context of laughter. Thus, it was felt much less likely that the patient displayed an epileptic seizure with laughter being one of the provoking factors.

He was then placed on carbamazepine. After this treatment, he had two episodes of mild staring but no frank seizures or immobilization.

His seizures have remained well controlled on this regimen for more than a year.

## Discussion

Smiles and laughter are universal human social gestures that involve a complex sequence of facial, pharyngeal and diaphragmatic muscle contractions and help to establish a friendly interaction with other people [1,2]. Several regions of the brain are associated with laughing. Laughter consists of an affective and a motor component. The emotional aspects are processed in the temporal lobe, whereas motor features are processed in frontal cortex [2]. The present data suggest that pericingulate premotor areas are involved in the triggering of the motor component of the laughter [3]. Reported cases reveal a high likelihood of cingulate and basal temporal cortex contribution to laughter and mirth in humans, and suggest the possibility that the anterior cingulate region is involved in the motor act of laughter, while the basal temporal cortex is involved in the processing of laughter’s emotional content in man [4]. Studies have shown that a small area on the left superior frontal gyrus, when stimulated consistently, produces laughter [4].

Normal laughter is a human behavioral response to pleasant feeling whereas pathological laughter is disproportionate to the emotional context [5]. Pathological laughter has been described in many clinical conditions including gelastic seizures and pseudobulbar palsy [4]. In many of the cases described so far, laughter was not associated with feelings of mirth [5]. However, there are a few case reports of the two occurring together [6,7]. The epileptogenic zone was circumscribed in the anterior and ventral part of the supplementary motor area and the underlying dorsal cingulate cortex. The symptomatogenic area for ictal laughter in the frontal lobe may reside in the superior frontal gyrus; however, substantial data are missing about the anatomic locations of frontal regions supporting gelastic seizures. Ictal laughter is the cardinal clinical sign of gelastic seizures in hypothalamic hamartomas and may also occur in extrahypothalamic epilepsy [8]. Focal brain lesions linked with gelastic seizure are generally located in the frontal or temporal region [9].

Gelastic seizures are most commonly described in patients with hypothalamic hamartoma causing precocious puberty [10]. An MRI scan dedicated to the hypothalamus, infundibulum, and mammillary bodies may yield a hamartoma as a cause of gelastic seizure [9]. Gelastic seizure associated with other types of lesions like focal cortical dysplasia is very uncommon and can usually be detected by high-resolution MRI but is difficult to localize with EEG [9,11]. Ictal EEG shows flattening of cerebral activity, especially if associated with hypothalamic hamartoma.

Our case was distinct from gelastic seizure as laughter actually induced seizure activities that were recorded and confirmed by two-day video EEG. Theoretically, if laughter were to trigger a seizure, the focus would be in the motor component (the pericingulate premotor area or anterior cingulate region), but this could not be confirmed on the basis of the video EEG of our patient. Due to the need to establish laughter as the causative agent in the seizures, video EEG is necessary to prove the temporal association and thus to confirm the diagnosis.

Gelastic seizure without anatomical lesion usually responds well to polytherapy with topiramate and carbamazepine, though most evidence is from case reports and small case series. If it is caused by hypothalamic hamartoma, stereotactic radiofrequency ablation provides a minimally invasive and low-risk approach compared with a direct surgical approach [8]. In our case, as no data were available in the literature, we started the patient on carbamazepine on top of the topiramate he was already on and laugh-provocation avoidance. He responded very well to the therapy. Further study is required to establish the standard treatment guidelines for this condition.

## Conclusions

Laugh-induced seizure remains a most unusual clinical entity, affecting quality of life most directly by laughing, which is an essential component of human life. Until February 2013, there was no case of laugh-induced seizure reported in the PubMed Central library, thus further study of this condition is required to guide clinicians in the proper management of the condition. As only limited data exist, clinicians must recommend a multimodal treatment, including a consideration of polytherapy and laugh-provocation avoidance.

### Patient’s perspective

Patients don’t want to be sick. They don’t make stuff up. It was frustrating for me to not be believed, to have multiple doctors tell me that all of my problems were psychiatric, just because I am bipolar. You know, people can have two diseases, as I turned out to have. It was very unusual for me to have bipolar symptoms and these symptoms, which had nothing to do with bipolar, and have people just tell me it was bipolar when I knew it wasn’t. So, my message to doctors is: ‘Believe your patients’.

## Consent

Written informed consent was obtained from the patient for publication of this case report, however, the patient does not wish the video of his EEG to be published. A copy of the written consent with the patient’s perspective is available for review by the Editor-in-Chief of this journal.

## Competing interests

The authors declare they have no competing interests.

## Authors’ contributions

NM and LJ were major contributors in writing the manuscript. MA, TS and MB edited the draft of the manuscript. RA did the final proofread of the manuscript, obtained patient consent and patient perspectives for the journal. All authors read and approved the final manuscript.
